# Extraction of Pyrrole from Its Mixture with *n*-Hexadecane Using Protic Ionic Liquids

**DOI:** 10.3390/molecules29174173

**Published:** 2024-09-03

**Authors:** Sorfina Amran, Muhammad Zulhaziman Mat Salleh, Hanee Farzana Hizaddin, Abdullah Amru Indera Luthfi, Noorashikin Md Saleh, Mohamed Kamel Hadj-Kali

**Affiliations:** 1 Department of Chemical and Process Engineering, Faculty of Engineering and Built Environment, Universiti Kebangsaan Malaysia, Bangi 43600, Malaysiaamru@ukm.edu.my (A.A.I.L.); noorashikin@ukm.edu.my (N.M.S.); 2 University of Malaya Centre for Ionic Liquids (UMCiL), University of Malaya, Kuala Lumpur 50603, Malaysia; hanee@um.edu.my; 3 Chemical Engineering Department, College of Engineering, King Saud University, P.O. Box 800, Riyadh 11421, Saudi Arabia

**Keywords:** denitrification, ionic liquid, protic ionic liquid, liquid–liquid extraction, NRTL, COSMO-RS, GAUSSIAN 09

## Abstract

The removal of nitrogen compounds from fuel via the conventional method, which is hydrodenitrogenation, is costly and involves catalysts and energy-intensive conditions (600 K and 300 atm). Recently, ionic liquids (ILs) have emerged as a promising alternative solvent for the denitrogenation of fuel oil. However, certain ILs are expensive and challenging to synthesize, prompting the exploration of protic ionic liquid (PIL) substitutes, which offer similar advantages to ILs. This study utilized the conductor-like screening model for real solvents (COSMO-RS) to predict the phase equilibria for three PILs—triethylammonium *p*-toluenesulfonate (TEA-TSA), triethylammonium salicylate (TEA-SA) and triethylammonium benzoate (TEA-BZ)—which were subsequently validated through experimental investigations. Liquid–liquid extraction experiments were conducted at 298 K and 1 atm, with pyrrole (serving as the model nitrogen compound) concentrations in *n*-hexadecane (representing the model fuel) ranging from 10 to 50 wt%. Additionally, the NRTL model effectively correlated the experimental tie lines. The obtained data indicated that TEA-TSA exhibited superior selectivity and distribution ratio compared to TEA-SA and TEA-BZ. All the ternary systems tested displayed positive slopes, suggesting a higher affinity of nitrogen compounds for the PIL. Supporting this observation, interaction energy (Δ*E*) and excess enthalpy (*H^E^*) were employed. The predicted outcomes revealed that TEA-TSA had high Δ*E*, and all PILs exhibited negative values of *H^E^.* The *H^E^* calculation underscored the significance of strong hydrogen bond interactions between pyrrole and the PIL for successful extraction.

## 1. Introduction

The investigation into producing fuels with significantly reduced levels of sulfur, nitrogen and aromatic content has gained widespread attention and scrutiny in response to the relentless escalation in environmental demands [1]. The release of harmful substances like nitrogen oxides (NOx), sulfur oxides (SOx), carbon monoxide (CO) and unburned hydrocarbon particles from gasoline or diesel engines has long been acknowledged as a major cause of urban air pollution. This issue has a profound impact on human health, the environment and economic progress [2]. Nitrogen-containing compounds often encountered in fossil fuels encompass nitriles, amines and heterocyclic aromatic substances, such as pyridine, indole and pyrroles [3]. Nitrogen-containing compounds impose limitations in the realm of oil upgrading and refining, affecting process operation, catalyst selection and process development [4]. In the industrial setting, hydrodenitrogenation (HDN) has conventionally served as the standard method for reducing the nitrogen content in fossil fuels. HDN entails a reduction process conducted in the presence of hydrogen and a catalyst, leading to the generation of NH_3_ (ammonia). Nevertheless, HDN still faces several limitations, including the requirement for high temperature and pressure conditions, substantial consumption of hydrogen and energy and the use of expensive catalysts [5]. In addition, HDN technology is capable of denitrifying saturated compounds on an industrial scale effectively. However, it encounters challenges when dealing with unsaturated aromatic compounds like pyridine, quinoline, indole, carbazole and their alkylated derivatives due to steric hindrance. This hindrance makes it difficult for these compounds to undergo conversion effectively on the catalyst surface. As of the present time, numerous non-HDN techniques have been suggested for the reduction in nitrogen content in fossil fuels. These limitations underscore the need for alternative denitrogenation methods that are more sustainable and cost-effective.

These methods encompass liquid–liquid extraction oxidation, distillation and adsorption. Among the array of denitrification methods mentioned, liquid–liquid extraction (LLE) has emerged as the subject of extensive scientific investigation. This method has garnered considerable interest due to its remarkable extraction efficiency, ease of operation and cost effectiveness. However, with LLE, particularly in the context of extractive denitrification (EDN), the selection of an appropriate solvent plays a crucial role in achieving the desired denitrification outcome. Conventional solvents like formamide [6], dimethylnaphthalene mixture [7], N,N-dimethylformamide [8] and acidic solvents [9] are known to have a negative environmental impact, which raises concerns and necessitates the exploration of more environmentally friendly alternatives for denitrification processes. In this context, ionic liquids (ILs) have emerged as promising alternatives due to their unique properties, such as low vapor pressure, high thermal stability and the ability to dissolve a wide range of compounds. To fulfil the demand for an extractive solvent with high capacity and selectivity for extracting aromatic nitrogen species, ionic liquids (ILs) present themselves as a superior alternative to conventional solvents. The unique properties of ILs, including low vapor pressure, high solubility for diverse organic and inorganic compounds and immiscibility with fuel, make them particularly well suited for this purpose. The majority of denitrogenation investigations utilizing ionic liquids have predominantly centered around model oils, serving as essential testbeds to explore the intricate chemistry and selectivity involved in the process. For instance, studies have showcased the impressive performance of dicyanamide-based ionic liquids in efficiently eliminating carbazole and pyridine from model oils [10].

Furthermore, employing diverse anions like BF_4_^−^, PF_6_^−^ and chloride-based ionic liquids (ILs) alongside conventional aromatic cations, such as imidazolium and pyridinium, has shown encouraging results in selectively extracting nitrogen-containing compounds from fuel oils. However, it is important to acknowledge that certain ILs in this scenario may present limitations, including the potential for generating corrosive hydrogen fluoride through the breakdown of fluorinated anions, elevated viscosity and susceptibility to air/moisture. To address these challenges, protic ionic liquids (PILs), a subclass of ILs, offer a compelling and intriguing alternative. PILs are readily synthesized by combining a Brønsted acid with a Brønsted base. In addition, PILs exhibit distinctive properties that make them highly sought after for applications as solvents or catalysts in various chemical reactions. Unlike aprotic ILs, PILs possess hydrogen ions attached to the cation, enabling the formation of hydrogen bonds that enhance solubility and specific interactions with substrates.

PILs offer several advantageous features, including affordability, low viscosity, ease of synthesis, purification and eco-friendliness. Indeed, protic ionic liquids (PILs) represent a relatively new and emerging class of ILs, and their applications in various research fields are still in the early stages. Consequently, the performances of PILs in different applications, including denitrogenation in fuel oils, have not been extensively studied. The limited research in this area can be attributed to a lack of awareness and understanding of PILs among researchers and industry professionals. Due to the wide range of available ionic liquids, studies on their effectiveness for nitrogen extraction are limited, and the synthesis of each protic ionic liquid can be costly. In addition, certain PILs are less chemically stable and prone to dissociation under certain conditions. To overcome this challenge, COSMO-RS emerges as a powerful tool for predicting the behavior, including chemical stability, likelihood of dissociation and optimizing the performance of these ionic liquids, reducing the need for expensive experimental synthesis. Cao et al. (2021) conducted an extensive investigation to evaluate the effectiveness of COSMO-RS in predicting Inγ^∞^ for ionic liquids (ILs) in extracting artemisinin from *Artemisia annua* L. A total of 903 ILs were predicted, and 14 of these ILs were selected for experimental validation, and the result showed that COSMO-RS predictions matched well with the experimental results. They found that artemisinin’s solubility depends on the anion and hydroxyl groups on the cation, and high extraction efficiency is achieved through hydrogen bonding between the solvent and solute [11]. Araya-López et al. (2022) used COSMO-RS to evaluate bistriflimide-based ionic liquids for separating levulinic and formic acids. They found that the solubility of both acids in the trihexyltetradecylphosphonium bistriflimide [P_66614_][Tf_2_N] was low, which matched COSMO-RS predictions. This low solubility is due to steric hindrance from the alkyl chains on the phosphonium cation, which blocks interactions between the phosphorus and oxygen atoms in the acids [12]. COSMO-RS is widely used across various industrial sectors due to its ability to model the diverse properties of ionic liquids (ILs) in extraction processes.

In this work, the extraction ability of triethylammonium with different anions, which are p-toluenesulfonate, salicylate and benzoate, in the removal of pyrrole from *n*-hexadecane is investigated. This approach offers a potential pathway for more efficient nitrogen removal processes, reducing the dependency on traditional HDN methods. The choice of the protic ionic liquid (PIL) TEA-TSA was based on a previous study by Fan et al. (2019), which demonstrated its effective extraction capabilities for quinoline, indole and carbazole [13]. In addition, the extraction efficiency after five regeneration cycles remains unchanged via back extraction. This makes it a promising candidate for predicting its extraction capability for pyrrole, a smaller compound, using COSMO-RS. Additionally, a study showed that anions with aromatic compounds, such as benzoate and salicylate, can enhance selectivity via π–π interaction [10]. An experimental investigation is conducted to validate the conductor-like screening model for real solvents (COSMO-RS) prediction in phase equilibria of a PIL–pyrrole–*n*-hexadecane system. Meanwhile, a non-random two-liquid model (NRTL) is utilized to correlate the experimental data.

## 2. Results and Discussion

### 2.1. Intermolecular Interactions

#### 2.1.1. Sigma Profile

**Figure 1:**
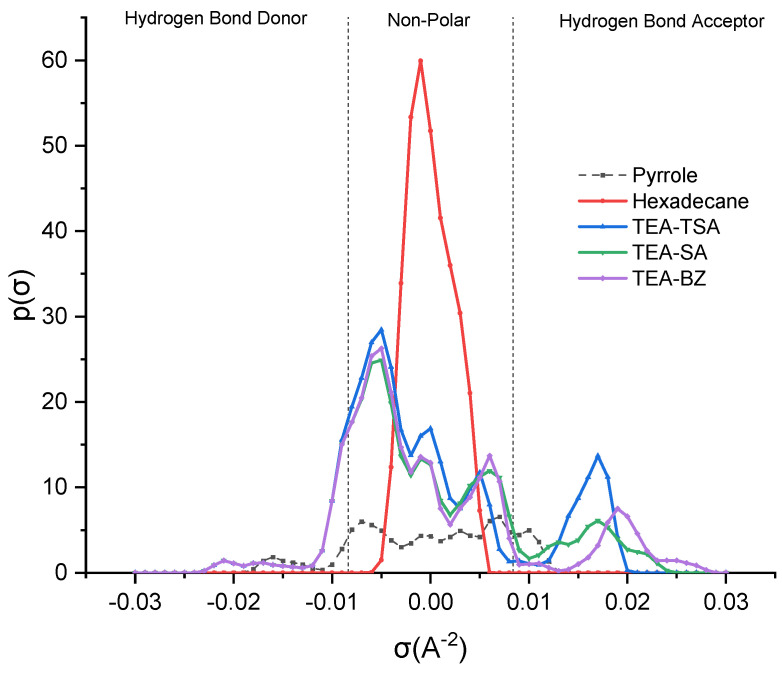
Sigma profile of PILs and its mixture.

In COSMO-RS analysis, sigma (σ) profiles provide insights into the molecular polarity, enabling predictions regarding the strength of interactions among different molecules [14]. Additionally, these profiles delineate regions such as the hydrogen bond donor region (HBD) (σ < −0.0084 e·Å^−2^), the non-polar region (−0.0084 e·Å^−2^ < σ < 0.0084 e·Å^−2^) and the hydrogen bond acceptor region (HBA) (σ > 0.0084 e·Å^−2^) [15]. The sigma profile of the mixture is depicted in Figure 1 below.

Based on the figure above, it is evident that the polarity of hexadecane is non-polar, stemming from its symmetric and linear structure, as well as the nature of carbon–carbon and carbon–hydrogen bonds [15]. Next, pyrrole demonstrates the ability to act as both a HBA and a HBD; thus, it can donate a hydrogen atom from the hydrogen attached to nitrogen and accept hydrogen bonds with its lone pair of electrons, but the prominent peak (−0.01821 e·Å^−2^) indicates the fact that it acts as an HBD compared to HBA (0.01337 e·Å^−2^). Thus, in order to interact with pyrrole, the PILs should exhibit both capabilities. In reviewing the PILs, it becomes evident that they have the potential to function as both a HBD and a HBA. Nevertheless, the prevailing trend leans toward HBA, likely influenced by the negative charge present on the sulfonate group and carboxylate group [14]. Furthermore, TEA-BZ appears more polarized compared to TEA-TSA and TEA-SA, suggesting a higher capacity to extract pyrrole. This shows that anions play a pivotal role compared to cations, which can be proven in the sigma profiles in Supplementary Materials, supporting the notion that the distribution peak of TEA predominantly falls in the non-polar region, with only a marginal presence in the hydrogen bond donor (HBD) area, indicating that TEA forms weak hydrogen bonds with pyrrole. In contrast, the anion is more polarized in the hydrogen bond acceptor (HBA) zone, forming stronger hydrogen bonds and significantly improving the extraction process.

#### 2.1.2. Sigma Potential

**Figure 2:**
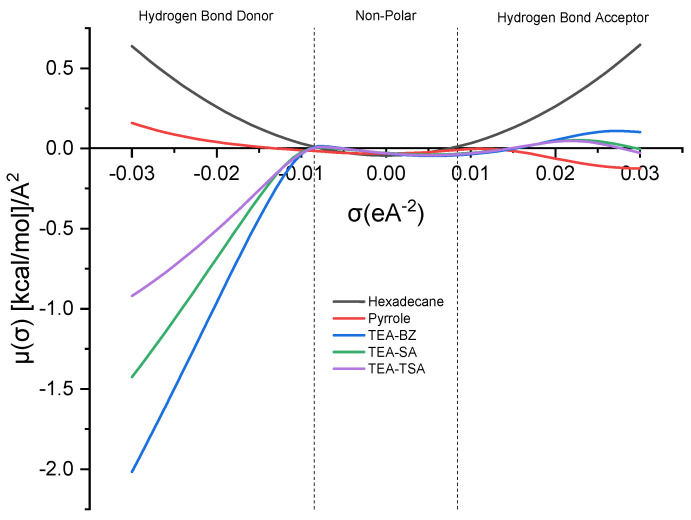
Sigma potential of PILs and its mixture.

The sigma (σ) potential acts as a gauge of the solvent’s inclination to interact with other elements in the mixture. In the σ-potential plot, a more negative value of μ (σ) indicates heightened attraction between molecules, whereas a greater positive value implies an escalation in repulsive tendencies [14,15]. Figure 2 below illustrates the sigma profile of the mixture.

The σ-potential curve of hexadecane demonstrates a parabolic shape, whereas pyrrole exhibits near symmetrical characteristics, with a slight inclination toward the HBA region. This observation indicates disparities in the sigma profile, where the peak is more pronounced toward the HBD region. All PILs show a negative value of µ(σ) in the HBD region, suggesting that there is a strong affinity between PILs and pyrrole to form a weak hydrogen bonding, with an order of TEA-BZ > TEA-SA > TEA-TSA.

### 2.2. Experimental Validation

#### 2.2.1. NMR Characterization

The chemical structures of the three PILs were identified using an NMR 400 MHz Bruker spectrometer: TEA-TSA: δ 1.120–1.124 ppm (t, 9H), 2.3 (s, 3H), 3.02–3.08 ppm (m, 6H), 6.42 ppm (s, 1H), 7.11–7.27 ppm (d, 2H), 7.67–7.7 ppm (d, 2H); TEA-SA: 1.35–1.39 ppm (t, 9H), 3.15–3.21 ppm (m, 6H), 6.27–6.28 ppm (m, 1H), 6.81–6.85 ppm (m, 2H), 6.91–6.94 ppm (m, 1H), 7.32–7.36 (m, 1H), 7.92–7.95 ppm (m, 1H); TEA:BZ: 1.28–1.35 ppm (t, 9H), 3.14–3.2 ppm (m, 6H), 6.27–6.29 ppm (s, 1H), 7.4–7.45 ppm (m, 2H), 7.47–7.52 ppm (m, 2H), 8.09–8.13 ppm (m, 2H).

#### 2.2.2. Ternary Liquid–Liquid Equilibrium

**Figure 3:**
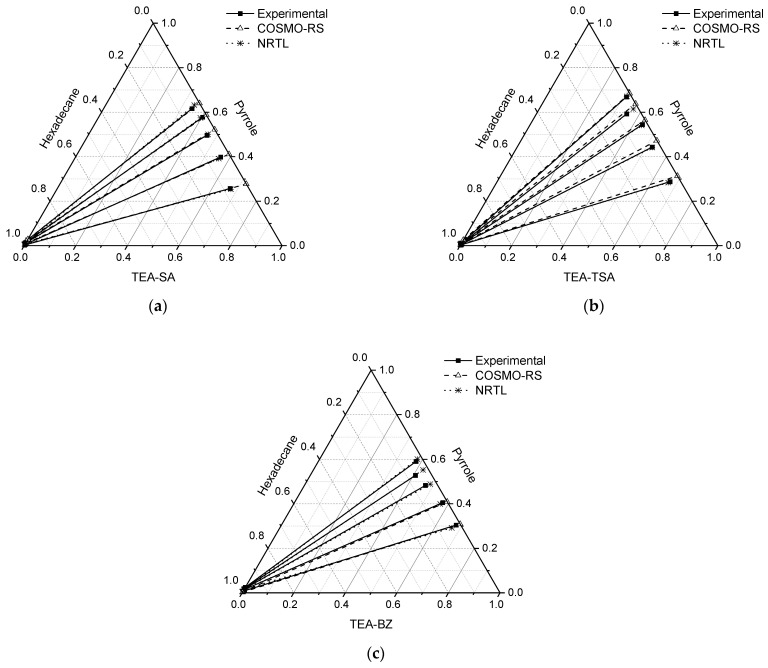
Tie lines for the ternary systems: (**a**) TEA-SA (1) + pyrrole (2) + *n*-hexadecane (3), (**b**) TEA-TSA (1) + pyrrole (2) + *n*-hexadecane (3) and (**c**) TEA-BZ (1) + pyrrole (2) + *n*-hexadecane at T = 298.15 K and 1 atm.

The LLE for the ternary system of PILs + pyrrole + hexadecane is displayed in Figure 3, and the molar compositions of the tie lines, distribution ratio (*D*), selectivity (*S*) and extraction efficiency (*E*) are tabulated in Table 1.

In all the ternary systems, a positive slope is observed, suggesting that nitrogen compounds in the extract phase exhibit a stronger affinity for the PIL mixture. This underscores the favorable attributes of the extracting solvent, as less solvent is necessary for nitrogen compound extraction. Moreover, the slope of the tie line in systems comprising TEA-SA and TEA-BZ was marginally lower compared to systems containing TEA-TSA. This suggests that as the concentration of pyrrole increases, the capacity of PILs for extraction decreases. Through a comparison with our previous findings involving aprotic ionic liquids and their binary mixture with the same model fuel oil [15], it becomes evident that PILs demonstrate a superior ability to extract pyrrole compared to aprotic ILs.

In addition, the results are consistent with the findings of Fan et al. (2019), which showed that TEA-TSA has a higher extraction efficiency for carbazole and indole compared to aprotic IL [C_4_mim]Cl, likely due to stronger π–π interactions between the non-basic nitrogen compounds and TSA [13]. Another comparison can be made with the study by Lemaoui et al. (2021) using deep eutectic solvents (DES), such as TPABr:AA, in the model fuel of *n*-decane. Their LLE results show that the gradient slope of the tie line is lower, indicating a lower capacity to extract pyrrole compared to our study. Additionally, the study by Hizaddin et al. (2016) on the extraction of pyrrole using TBAB/EG-type DES in a model fuel of *n*-hexadecane demonstrates that as the concentration of pyrrole increases, the effectiveness of the DES also increases, showing superior selectivity compared to our study. However, in terms of capacity, our study exhibits greater performance, as evidenced by the gradient of the tie line slope [16].

#### 2.2.3. COSMO-RS Prediction vs. Experiments

Figure 3 indicates that COSMO-RS fails to accurately predict the series tie line within the concentration range of 0.6–0.1 g (pyrrole) for the TEA-BZ system. Upon examining the raffinate phase across all systems, COSMO-RS demonstrates satisfactory accuracy in predicting experimental outcomes in the absence of PILs. However, analysis of the extract phase reveals the experimental presence of hexadecane, contrary to COSMO-RS predictions. Nonetheless, the observed quantity of hexadecane remains as low as 0.1 mol, as detailed in Table 1. Regarding the slope of the series tie line, the TEA-BZ system at concentrations ranging from 0.2 to 0.4 and TEA-SA consistently aligns with COSMO-RS predictions. Conversely, for the TEA-TSA system, COSMO-RS predicts a slightly higher slope. Consequently, it can be inferred that all systems exhibit positive slopes, indicating the favorable affinity of PILs for pyrrole.

#### 2.2.4. Distribution Ratio and Selectivity

**Figure 4:**
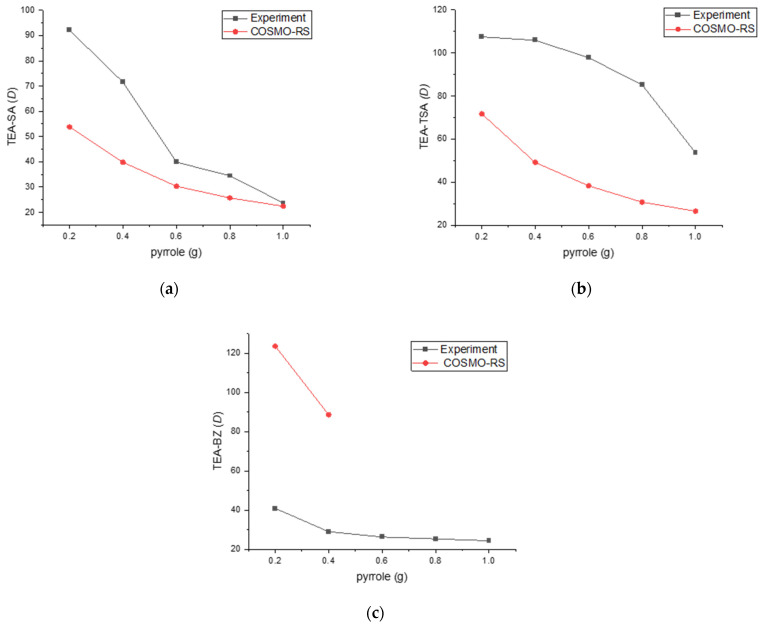
Experimental distribution ratio vs. COSMO-RS for (**a**) TEA-SA, (**b**) TEA TSA and (**c**) TEA-BZ systems with *n*-hexadecane as a model fuel.

**Figure 5:**
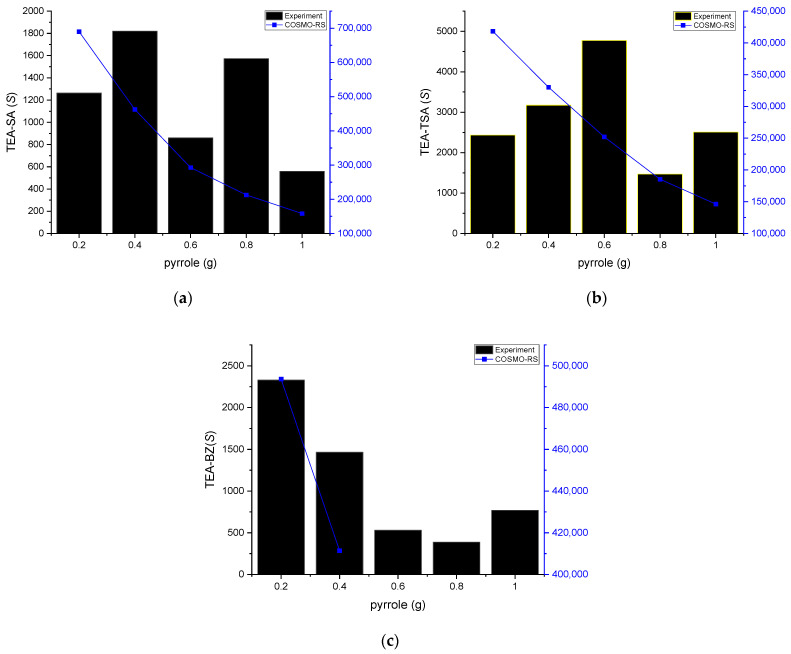
Experimental selectivity vs. COSMO-RS of (**a**) TEA-SA, (**b**) TEA TSA and (**c**) TEA-BZ with *n*-hexadecane as a model fuel.

Table 1, Figure 4 and Figure 5 provide insights into the distribution ratio and selectivity based on the concentration of nitrogen compounds. The distribution ratio assesses the PILs’ capacity to extract nitrogen compounds from hexadecane, while selectivity measures the efficiency of extracting nitrogen compounds while minimizing the extraction of hexadecane.

As displayed in Figure 4, the experimental distribution ratio for the TEA-TSA and TEA-SA systems was higher as compared to COSMO-RS prediction, while TEA-BZ was shown to be different and failed to predict its *D* in 0.6–1 g of pyrrole concentrations. The arrangement of *D* values for pyrrole, based on the solvent employed, is as follows: TEA-TSA > TEA-SA > TEA-BZ. The high value of *D* for TEA-TSA is due to the size of the anion, which causes less steric hindrance, resulting in an ease in the mobility of pyrrole in binding with the PILs. In addition, the low correlation of prediction between COSMO-RS and experimental data in the distribution ratio or even in the tie line could be the parameter limitation. This is because the accuracy of COSMO-RS predictions depends on precise parameterization, such as dielectric constants and surface areas, which may not perfectly reflect the actual experimental condition. This assertion can be supported by a previous study by Wang et al. (2014); in predicting LLE in systems containing DES, the use of TZVP and TZVP FINE parameters shows that COSMO-RS is more effective in describing the non-ideal behavior of systems containing alcohols compared to those with aromatics and cyclic compounds, likely due to the intricate interactions present in aromatic and cyclic systems [17]. It can be observed that COSMO-RS qualitatively predicted similar trends of correlation between the distribution ratio and the molar composition of pyrrole.

PILs with high selectivity for nitrogen compounds are beneficial, as they entail fewer stages needed to remove them, resulting in lower capital costs. As seen in Figure 5, COSMO-RS predicted high selectivity as compared to experimental data, with selectivity gradually decreasing as the concentration of pyrrole increased. However, the experimental data on selectivity present a fluctuating graph, which leads to a lack of discernible trend as the pyrrole concentration increases. The experimental trend is the same as in our previous study using aprotic ionic liquid [EMIM]NCN_2_ [15] and [EMIM]MESO_3_ [18], but the selectivity is much higher compared to the PILs. Anions play a pivotal role in PIL selectivity toward pyrrole, as evidenced by the sigma profile, highlighting the heightened polarization and robust hydrogen bonding in their presence [19]. Moreover, the contribution to enhanced selectivity can be discerned through the partial charge on the anion heteroatom [20]. Atomic charge calculation was employed in this study [21], revealing the following partial charges: benzoate (−0.4943, −0.8093), salicylate (−0.8464, −0.9902) and p-toluenesulfonate (−0.9754, −0.5308, −0.9773). Notably, the total charge for p-toluenesulfonate surpassed other anions, resulting in heightened selectivity for pyrrole extraction. Regarding reusability, PILs are known for their tunable properties, which can allow for reversible interactions with the target compounds, such as pyrrole. After the extraction process, pyrrole can potentially be removed from the PIL by adjusting external conditions, such as temperature and pressure, or by introducing a back-extraction solvent that has a higher affinity for pyrrole than the PIL.

### 2.3. Interaction Energy

The solubility of a solute in an ionic liquid is contingent upon various factors, such as the interactions between the solute and the ions present in the ionic liquid. These interactions can be influenced by the size, shape and charge distribution of both the solute and the ions in the ionic liquid. The proximity of the cation and anion in an ionic liquid can influence the intensity and character of the interactions between the ions and solute molecules [22]. When the cation and anion are closely aligned in the ionic liquid, the interactions between ions and solute molecules may be enhanced, potentially resulting in increased solubility of heterocyclic nitrogen compounds. On the contrary, when the cation and anion are spaced further apart in the protic ionic liquid, the interactions between ions and solute molecules may weaken, making it easier for pyrrole to be accommodated between the PILs [22,23,24]. In this study, the calculation of ion exchange (IE) was conducted using the following equation:

(1) $$∆E=E_{IL\left(Complex\right)-}{(E}_{Cat}+E_{An})$$

Let $E_{IL}$ represent the total energy of an ionic liquid, while $E_{Cat}$ and $E_{An}$ denote the total energy of the cation and anion, respectively [24]. Negative values of IE signify favorable interactions between the molecules in the system. This suggests that minimal energy is needed to dissociate the molecule or complexes [24]. Table 2 below illustrates the interactions within the IL.

The table presented above illustrates negative energy values for all PILs, suggesting favorable interactions among the molecules, with TEA-TSA exhibiting the highest interaction energy (IE), followed by TEA-TSA and TEA-BZ. A high IE value in TEA-TSA implies greater solubility of pyrrole and enhanced selectivity compared to other PILs. Conversely, TEA-BZ displays a weaker IE, indicative of a less compact structure. This characteristic facilitates easier restructuring of the cation and anion, enabling better accommodation of pyrrole molecules [22]. Pyrrole is known to act as both a hydrogen bond donor and acceptor, which could facilitate interaction with the TEA cation. Specifically, the lone pair of electrons on the nitrogen atom in pyrrole could interact with the hydrogen attached to the nitrogen in TEA, potentially leading to the formation of a new PIL. It is suggested that while the formation of pyrrole-based PILs is thermodynamically feasible, the extent of this reaction is likely limited under the experimental conditions used, given the weak acidity of pyrrole. Additionally, any such formation would be in equilibrium with the original PILs and would not significantly alter the overall extraction efficiency observed in our experiments. Further studies could be conducted to quantitatively assess the extent of this reaction.

### 2.4. Excess Enthalpy

The excess enthalpy determined using the COSMO-RS model reflects the intermolecular interactions (including misfit, hydrogen bonds and van der Waals energies) between the pyrrole and PILs [25]. The excess enthalpy (*H^E^*) of a mixture can be predicted using the following equation:

(2) $$H_{m}^{E}=\sum x_{i}H_{m}^{E}=\sum x_{i}[H_{\left(i,mixture\right)-}H_{\left(i,pure\right)}]$$

where $H_{m}^{E}$ denotes the excess enthalpy of individual molecules within the mixture, indicating the deviation in enthalpy between component iii in the mixture and its pure state. Conversely, the excess enthalpy of a mixture can be described as the aggregate of three factors (as per Equation (3)): electrostatic misfit, hydrogen bonding and van der Waals interactions.

(3) $$H_{M}E=H_{M}E\left(misfit\right)+H_{M}E\left(Hbond\right)+H_{M}E(vDw)$$

**Figure 6:**
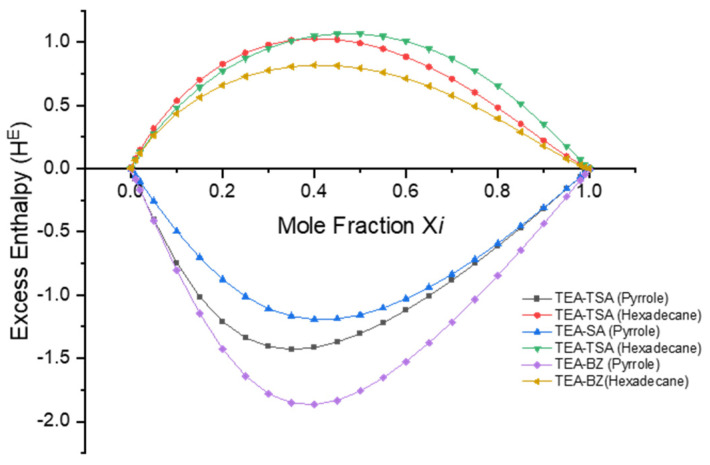
Excess enthalpy value of hexadecane and pyrrole in their binary mixtures with each PILs.

**Figure 7:**
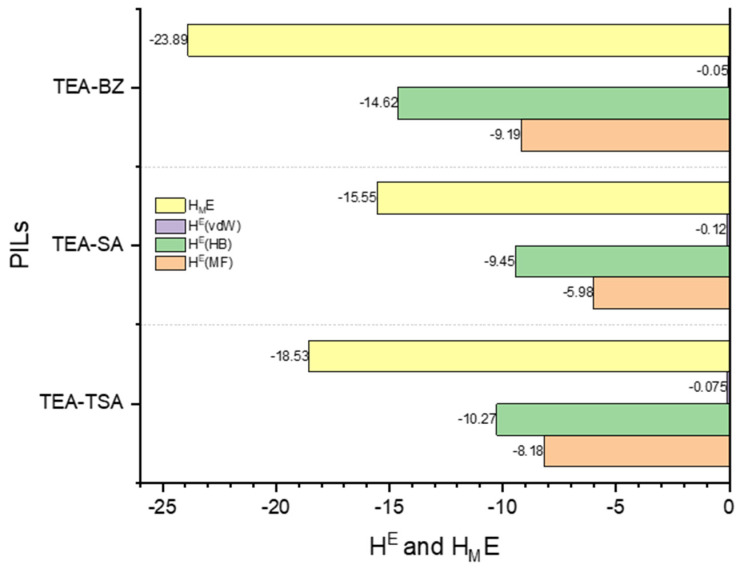
Energetic contributions to the excess enthalpy of mixtures between PILs and pyrrole were forecasted by COSMO-RS at a temperature of 298.15 K.

As previously mentioned, excess enthalpy reflects the intermolecular interactions, including misfit, hydrogen bonding and van der Waals energies, between the pyrrole and PILs. These interactions were computed using the COSMO-RS model and are illustrated in Figure 6 and Figure 7 below.

Based on Figure 6, it is demonstrated that the negative excess enthalpy observed when pyrrole is dissolved in a PIL compared to hexadecane indicates that the interactions between pyrrole and the PILs are more energetically favorable in the order of TEA-BZ > TEA-TSA > TEA-SA. This suggests that there are specific attractive interactions between the pyrrole molecules and the ions present in PILs. These interactions could include hydrogen bonding between the PILs and pyrrole molecules, which contribute to the stabilization of the mixture. Conversely, the positive excess enthalpy observed when pyrrole is dissolved in hexadecane suggests that the interactions between pyrrole and hexadecane are less favorable than expected. This could be due to differences in the polarity and molecular structure between pyrrole and hexadecane, leading to weaker interactions compared to those in PILs. This assertion can be proven in Figure 7; when the excess enthalpy (*H_M_^E^*) of pyrrole in PILs shows a negative value, this signifies an exothermic process, indicating that the dissolution of pyrrole in PILs is favorable [25]. The histogram depicted in Figure 7 illustrates that the hydrogen bond interaction energy between PILs exerts a more significant influence on the dissolution capability of pyrrole in PILs, followed by misfit and van der Waals interactions, with an order of TEA-BZ > TEA-TSA > TEA-SA. The strong hydrogen bonding between TEA-BZ and pyrrole can be proven through the sigma profile in which TEA-BZ is more polarized as compared to other PILs. Indeed, it can be inferred that anions play a crucial role in the extraction of pyrrole.

### 2.5. Electrostatic Potential (ESP)

**Figure 8:**
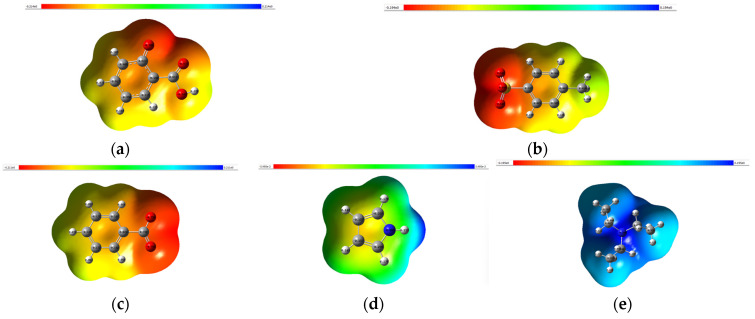
Electrostatic potential surfaces of anion: (**a**) SA, (**b**) TSA (**c**) BZ, (**d**) pyrrole and (**e**) TEA optimized at the B3LYP/6-311G + (2d,p) level.

ESP is often represented visually using color maps on molecular surfaces. In these representations, regions with highly negative ESP are typically depicted in red, indicating an attraction to positively charged species or electron-rich regions. Conversely, regions with highly positive ESP are usually depicted in blue, indicating an attraction to negatively charged species or electron-deficient regions. The ESP was calculated using Gaussian 09 via the surface and contours. Figure 8 shows the ESP surface and for a better resolution please refer to the Supporting Information.

The ESP can provide insights into hydrogen bonding interactions. Hydrogen bond donors typically exhibit regions of positive ESP (electron-poor), while hydrogen bond acceptors usually display regions of negative ESP (electron-rich). The electrostatic potential (ESP) distributions of SA, BZ, TSA, TEA and pyrrole provide crucial insights into their chemical properties and reactivity. SA and BZ benzoate share structural similarities, with both featuring carboxylate groups (-COO^−^) and aromatic rings. Consequently, they exhibit negative ESP around these functional groups, highlighting their electron-rich nature. The presence of the hydroxyl group (-OH) in salicylate further accentuates its negative ESP, offering potential sites for hydrogen bonding interactions.

In contrast, TSA showcases a distinct ESP pattern due to its sulfonate group (SO_3_^−^) attached to a toluene ring. The negative charge on the sulfonate group results in negative ESP, while the toluene ring contributes to an electron-rich region akin to an aromatic ring. TEA exhibits positive ESP primarily around the nitrogen atom owing to its positively charged ammonium ion (NR_3_^+^). The ethyl groups do not significantly influence the ESP distribution. Lastly, pyrrole presents a unique ESP profile characterized by negative ESP around the nitrogen atom and the aromatic ring. This negative ESP arises from the lone pair on nitrogen and the delocalized π electrons in the aromatic ring, emphasizing its electron-rich nature.

### 2.6. NRTL Modeling

The compositions for liquid–liquid equilibrium are derived by solving an isothermal liquid–liquid flash under predefined temperature and pressure conditions using the subsequent system of equations:

Material Balance:

(4) $$x_{i}-\left(1-\omega \right)x_{i}^{L1}-\omega x_{i}^{L2}=0,i=1,N_{c}$$

Equilibrium Equation:

(5) $$x_{i}^{L1}\gamma_{i}^{L1}-x_{i}^{L2}\gamma_{i}^{L2}=0,i=1,N_{c}$$

Equation of Summation:

(6) $$\sum_{i}x_{i}^{L1}-\sum_{i}x_{i}^{L2}=0$$

Here, ω represents the liquid–liquid splitting ratio; *x_i_* denotes the quantity of component *i* in the mixture; $x_{i}^{L1}$ represents the quantity of component *i* in liquid phase *L*1; $x_{i}^{L2}$ represents the quantity of component *i* in liquid phase *L*2; and *N_C_* represents the number of constituents of the liquid phases. The parameters $\gamma_{i}^{L1}$ and $\gamma_{i}^{L2}$ denote the activity coefficients of component *i* in *L*1 and *L*2, respectively. In this study, the activity coefficients were determined using the non-random two-liquid (NRTL) model [26]. For a system with multiple components, the activity coefficient of component *i* is calculated using the following general expression:

(7) $$\ln\gamma_{i}=\frac{\sum_{j}\tau_{ji}G_{ji}x_{j}}{\sum_{j}G_{ji}x_{j}}+\sum_{j}\frac{G_{ji}x_{j}}{\sum_{k}G_{kj}x_{k}}\left(\tau_{ij}-\frac{\sum_{k}\tau_{kj}G_{kji}x_{k}}{\sum_{k}G_{kj}x_{k}}\right)\;\mathrm{with}\;\ln G_{ij}=-\alpha_{ij}\tau_{ij},\alpha_{ij}=\alpha_{ji}\;\mathrm{and}\;\tau_{ii}=0$$

The binary interaction parameters *τ_ij_* and *τ_ji_* are used, along with *α_ij_*, the non-randomness parameter. A value of zero for *α_ij_* indicates an ideal solution, meaning it is completely random.

When the NRTL model was first derived, Renon and Prausnitz suggested that *α_ij_* was related to Guggenheim’s quasi-chemical approximation and was approached to a value between 0.1 and 0.3. Later, its value was corrected to between 0.2 and 0.47. For many systems, *α_ij_* = 0.2 resulted in an accurate fit for the ternary LLE systems with DESs and ILs. The same value was therefore retained in this work.

The thermodynamics model development in this research was conducted using the Simulis^®^ environment. The estimation of binary interaction parameters *τ_ij_* and *τ_ji_* was achieved by minimizing the root mean square deviation (RMSD). Table 3 displays the RMSD values for each system.

All RMSD values for the NRTL correlation fall below 2%, suggesting that the NRTL activity coefficient model accurately represents the experimental data. This alignment is further evident in the ternary plots, where the connecting lines calculated by NRTL coincide with the experimental connecting lines, indicating excellent agreement. The values of the binary NRTL interaction parameters fitted to each ternary system are detailed in Table 4.

To ensure consistency between this study and the previous one, which focused on the same binary system (pyrrole extraction from *n*-hexadecane), the binary interaction parameters between these two molecules were directly adopted from our prior work [22] without modification. This approach minimizes the number of interaction parameters and allows the obtained data to be utilized in any process simulation software for subsequent design purposes.

## 3. Materials and Methods

### 3.1. Experimental Methodology

The chemicals utilized in this study are outlined in Table 5 and were used in their as-received state without further purification. To ascertain the ternary composition of the extract and raffinate phases at equilibrium, nuclear magnetic resonance (NMR) spectroscopy was employed, with deuterated chloroform (≥99.8%) serving as the solvent.

#### 3.1.1. Synthesis of PILs

The synthesis of protic PILs involves mixing equal moles of cations and anions at room temperature for 30 min to form a homogeneous liquid. The resulting product is then dried in an oven at 60 °C for 6 h to obtain a colorless liquid.

#### 3.1.2. LLE Experiment

**Figure 9:**
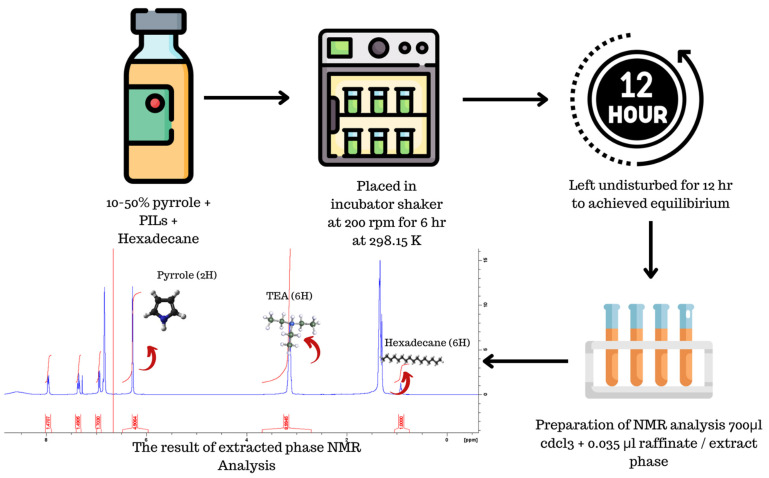
An illustration of the experimental methodology of the flow diagram.

The feed mixture was meticulously prepared within a 20 mL screw-capped scintillation vial, utilizing an analytical balance with a precision of ±0.0001 g. This mixture comprised various weight percentages (10, 20, 30, 40 and 50 wt%) of pyrrole in hexadecane, with the total weight standardized to 2 g. Furthermore, an ionic liquid (IL) was added to each vial in a 1:1 mass ratio. To prevent any loss of components due to evaporation, the vials were tightly sealed with parafilm, and spring clamps were applied during the shaking process. Shaking was carried out at 200 rpm in an incubation shaker maintained at a constant temperature of 298.15 K and atmospheric pressure of 1 atm. The mixing duration was set for 6 h. Subsequently, after the 6 h mixing period, the system was left undisturbed for an additional 12 h to ensure that equilibrium was attained. The decision to wait for 12 h was based on a settling time study conducted to ensure full equilibration within this time frame. Figure 9 below illustrates a flow diagram of the experimental methodology.

#### 3.1.3. Determination of Molar Composition

For the analysis of composition, a small sample volume (±0.035 mL) was extracted from both the extract and raffinate layers using a micropipette. To prevent potential contamination from the raffinate layer, a bubble was purged from the micropipette tip before withdrawing the sample from the extract layer. The extracted sample was then dissolved in 0.7 mL of deuterated chloroform and placed inside an NMR tube, ensuring a homogeneous mixture of sample and solvent. Each NMR tube was tightly sealed with parafilm to prevent any chemical loss during analysis. The 1H NMR spectrometer from Bruker (Billerica, MA, USA version 4.1.4), operating at 400 Hz, was employed to measure the peaks of hydrogen molecules in each component of the sample, enabling the identification and quantification of substances in the extract and raffinate layers. The 1H NMR spectra of each component were obtained using an NMR 400 MHz Bruker spectrometer. Specific hydrogen peaks with chemical shifts in ppm were selected—TEA ± 3.0 multiplet (6H), pyrrole ± 6.7 singlet (2H), hexadecane ± 0.92 singlet (6H)—to determine the ternary composition. The molar fraction of each element in both layers was calculated using Equation (8).

(8) $$x_{i}=\frac{H_{i}}{\sum_{i=1}^{3}H_{i}}$$

where $H_{i}$ represents the hydrogen peak area in component ⅈ, while $x_{i}$ denotes the mole fraction of species ⅈ. The mole fraction average uncertainty across all experiments was estimated to be <0.003.

#### 3.1.4. Experimental Selectivity and Distribution

Assessment of the extraction efficiency was conducted using Equations (9) and (10), which utilize the distribution ratio of the nitrogen compound (D) and the selectivity (S) of each PIL.

(9) $$D_{p}=\frac{x_{p}^{1}}{x_{p}^{2}}$$

(10) $$S=\frac{x_{p}^{1}}{x_{p}^{2}}/\frac{x_{H}^{1}}{x_{H}^{2}}$$

The pyrrole and hexadecane concentrations are denoted with $x_{p}$ and $x_{H}$, respectively. The extract phase is identified as 1, while the raffinate phase is labeled as 2.

#### 3.1.5. Extraction Efficiency

The extraction efficiency was determined using Equation (11).

(11) $$E=\left(1-\frac{c_{w_{o}}}{c_{w}}\right)\times 100$$

*Cw*_o_ and *Cw* denote the concentrations of pyrrole in the raffinate and extract phases, respectively.

#### 3.1.6. COSMO-RS Prediction

All calculations were performed using the COSMOthermX19 software version 19.0.5 with the BP-TZVP C30_1401 parameterization. The data calculations were derived through the density functional theory (DFT), utilizing the Becke–Perdew (BP) functional alongside the resolution of identity (RI) approximation and a triple valence polarization (TZVP) basis set.

## 4. Conclusions

A comprehensive understanding of the role of protic ionic liquids (PILs) in the extraction of pyrrole remains limited, both in terms of simulation and experimental exploration. Hence, this study aimed to elucidate the extraction potential of PILs for pyrrole using the COSMO-RS model, validated through experimental investigation. The prediction of key thermodynamic properties, such as excess enthalpies (HE), and phase equilibria is crucial in assessing the extraction efficiency of various PILs. Analysis of the liquid–liquid extraction (LLE) results reveal a positive slope for the tie lines across all systems, indicating a strong affinity of nitrogen compounds for PILs, with TEA-TSA demonstrating the highest efficiency, followed by TEA-SA and TEA-BZ. This finding suggests that PILs require less solvent for extraction, thus reducing costs. This observation is further supported by interaction energy calculations, with TEA-TSA exhibiting notably high interaction energy, emphasizing its effectiveness. Moreover, hydrogen bond interactions between pyrrole and PILs are identified as significant contributors to the extraction process, followed by misfit and van der Waals interactions. Additionally, successful tie line correlation using the NRTL model is achieved in all ternary diagrams. Overall, this study proposes a novel and potentially cost-effective approach for denitrogenating fuel oil by employing PILs.

## Figures and Tables

**Table 1:** Experimental LLE result for systems containing TEA-TSA (1) + pyrrole (2) + *n*-hexadecane (3), TEA-SA (1) + pyrrole (2) + *n*-hexadecane (3) and TEA-BZ (1) + pyrrole (2) + *n*-hexadecane (3) at T = 298.15 K and P = 1 atm.

Extract			Raffinate			*D*	*S*	*E*%
**TEA-TSA (1) + Pyrrole (2) + *n*-Hexadecane (3)**								
X′1	X′2	X′3	X″1	X″2	X″3			
0.670	0.288	0.042	0.000	0.003	0.997	102.98	2433.93	99.03%
0.524	0.443	0.033	0.000	0.004	0.996	105.93	3173.81	99.06%
0.435	0.545	0.020	0.000	0.006	0.994	97.81	4774.11	98.98%
0.350	0.592	0.058	0.000	0.007	0.993	85.19	1467.14	98.83%
0.311	0.668	0.021	0.000	0.012	0.988	53.69	2508.73	98.14%
**TEA-SA (1) + Pyrrole (2) +*n*-Hexadecane (3)**								
0.670	0.257	0.073	0.000	0.003	0.997	92.14	1263.97	98.91%
0.563	0.398	0.039	0.000	0.006	0.994	71.54	1819.96	98.60%
0.460	0.496	0.044	0.000	0.013	0.987	37.95	860.63	97.37%
0.403	0.578	0.019	0.000	0.017	0.983	34.97	1763.99	97.14%
0.342	0.616	0.041	0.000	0.026	0.974	23.70	559.12	95.78%
**TEA-BZ (1) + Pyrrole (2) + *n*-Hexadecane (3)**								
0.678	0.305	0.017	0.000	0.007	0.993	40.93	2328.68	97.56%
0.575	0.406	0.019	0.000	0.014	0.986	28.98	1466.30	96.55%
0.475	0.476	0.049	0.000	0.018	0.982	26.28	530.29	96.19%
0.410	0.527	0.064	0.000	0.021	0.979	25.32	389.39	96.05%
0.379	0.590	0.031	0.000	0.024	0.976	24.35	769.99	95.89%

**Table 2:** Interaction energy values between IL complexes.

IL	Δ*E* IL (kcal/mol)
TEA-TSA	−1404.09
TEA-SA	−1085.10
TEA-BZ	−1019.08

**Table 3:** RMSD values for NRTL correlation and COSMO-RS prediction of ternary LLE systems.

System	RMSD (%)
[TEA][TSA] (1) + pyrrole (2) + *n*-hexadecane (3)	0.88
[TEA][SA] (1) + pyrrole (2) + *n*-hexadecane (3)	0.72
[TEA][BZ] (1) + pyrrole (2) + *n*-hexadecane (3)	1.30

**Table 4:** Values of NRTL binary interaction parameters for each ternary system.

i–j	$\tau_{ij}$	$\tau_{ji}$
pyrrole—[TEA][TSA]	881.2	−383.5
pyrrole—[TEA][SA]	992.1	−177.0
pyrrole—[TEA][BZ]	1062.4	107.6
*n*-hexadecane—[TEA][TSA]	2464.6	288.1
*n*-hexadecane—[TEA][SA]	2456.7	211.8
*n*-hexadecane—[TEA][BZ]	2397.8	363.3
pyrrole—*n*-hexadecane	1073.9	824.7

**Table 5:** Materials used in this experiment.

Chemical Name	CAS No.	Supplier	Purity (wt%)	Abbreviation
Triethylamine	121-44-8	Sigma Aldrich (St. Louis, MO, USA)	≥99.5	TEA
Salicylic acid	69-72-7	Sigma Aldrich	≥99%	SA
Benzoic acid	65-85-0	Sigma Aldrich	≥99.5%	BZ
P-Toluenesulfonic acid monohydrate ReagentPlus	6192-52-5	Sigma Aldrich	≥98.0	TSA
Pyrrole	109-97-7	Sigma Aldrich	99.0	Pyr
Hexadecane	544-76-3	Sigma Aldrich	99.0	Hexa
Chloroform-d	865-49-6	Sigma Aldrich	99.8	CDCl3

## Data Availability

Data are contained within the article and Supplementary Materials.

## Supplementary Materials

The following supporting information can be downloaded at: https://www.mdpi.com/article/10.3390/molecules29174173/s1, Figure S1: The Sigma Profile of Cation and Anion, Figure S2: Electrostatic Potential Surfaces of Anion: (a) SA, (b) TSA (c) BZ, (d) pyrrole and (e) TEA Optimized at the B3LYP/6-311G + (2d,p) level.
