# Supplementary material for: Extraction of Pyrrole from Its Mixture with n-Hexadecane Using Protic Ionic Liquids
Source: Molecules. 2024 Sep 3;29(17):4173. doi: 10.3390/molecules29174173 (PMC11397634; doi:10.3390/molecules29174173)
Supplement: Supplementary file 1 [file molecules-29-04173-s001.zip › molecules-3139387-supplementary.pdf]

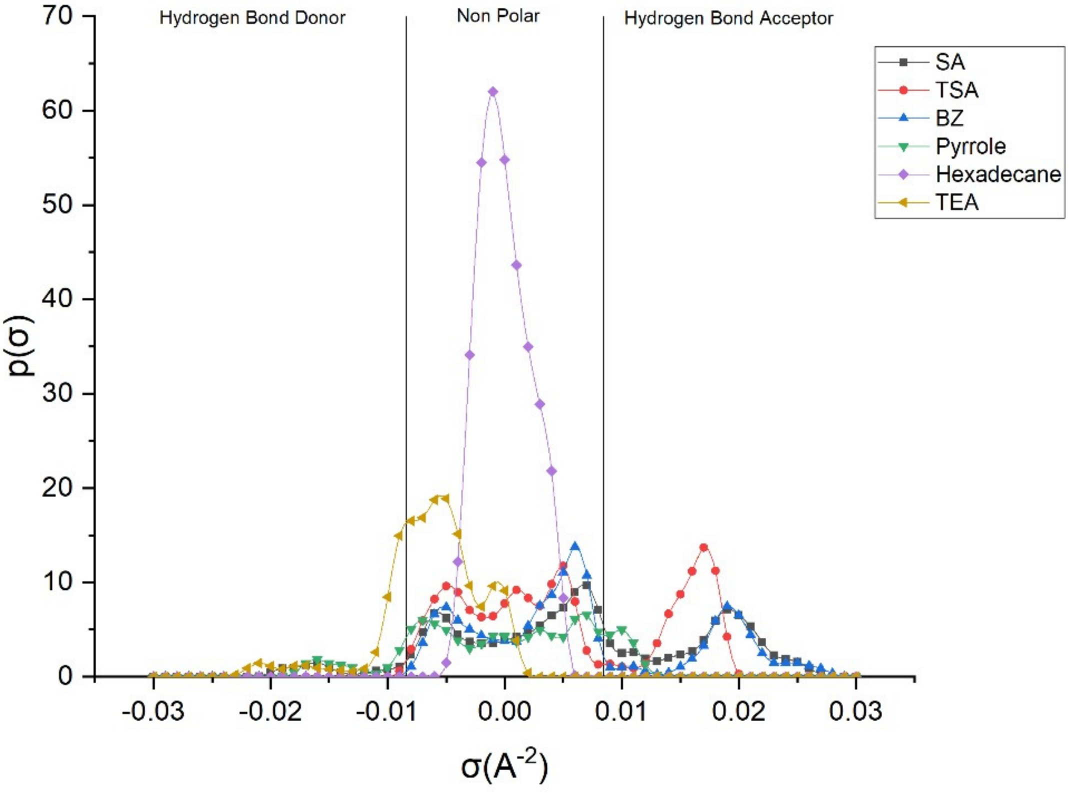

Figure S1 The Sigma Profile of Cation and Anion

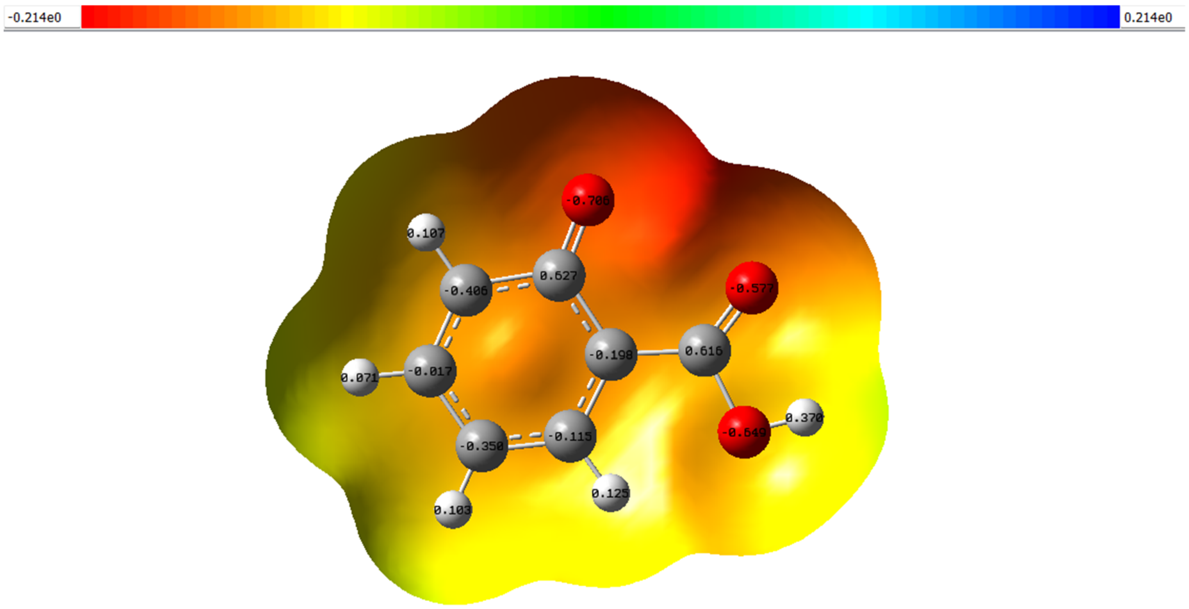

(a)

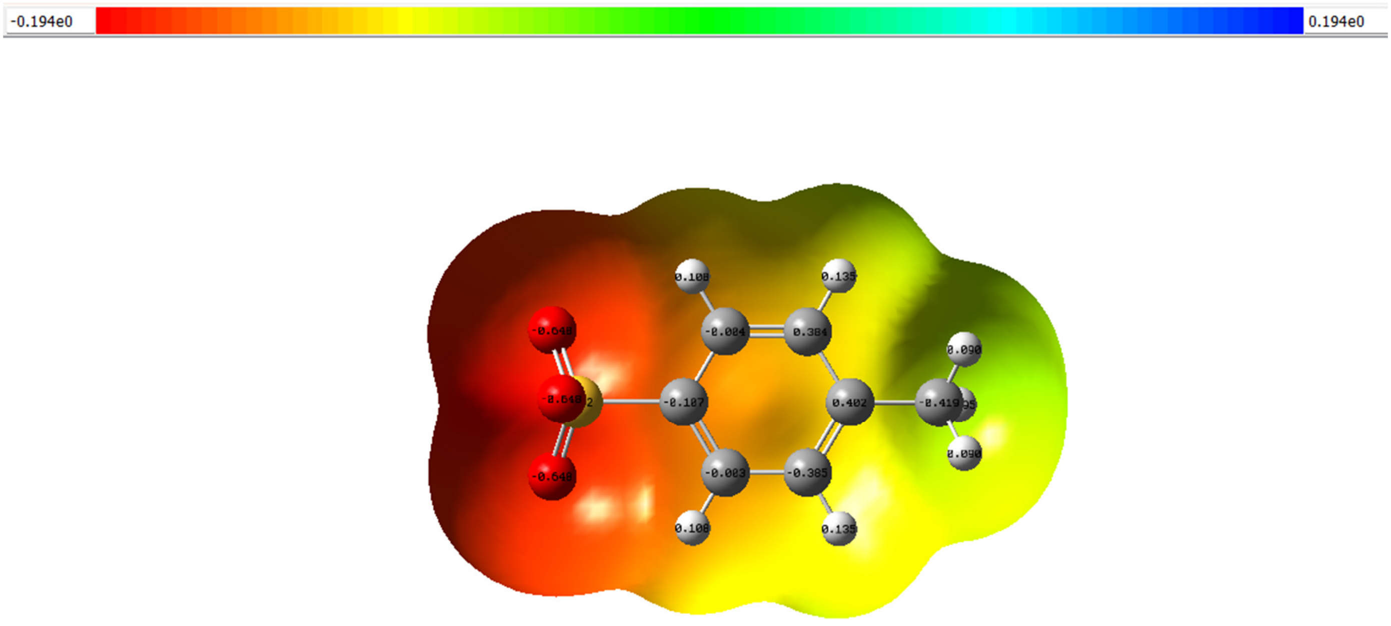
-0.194e0
0.194e0

(b)

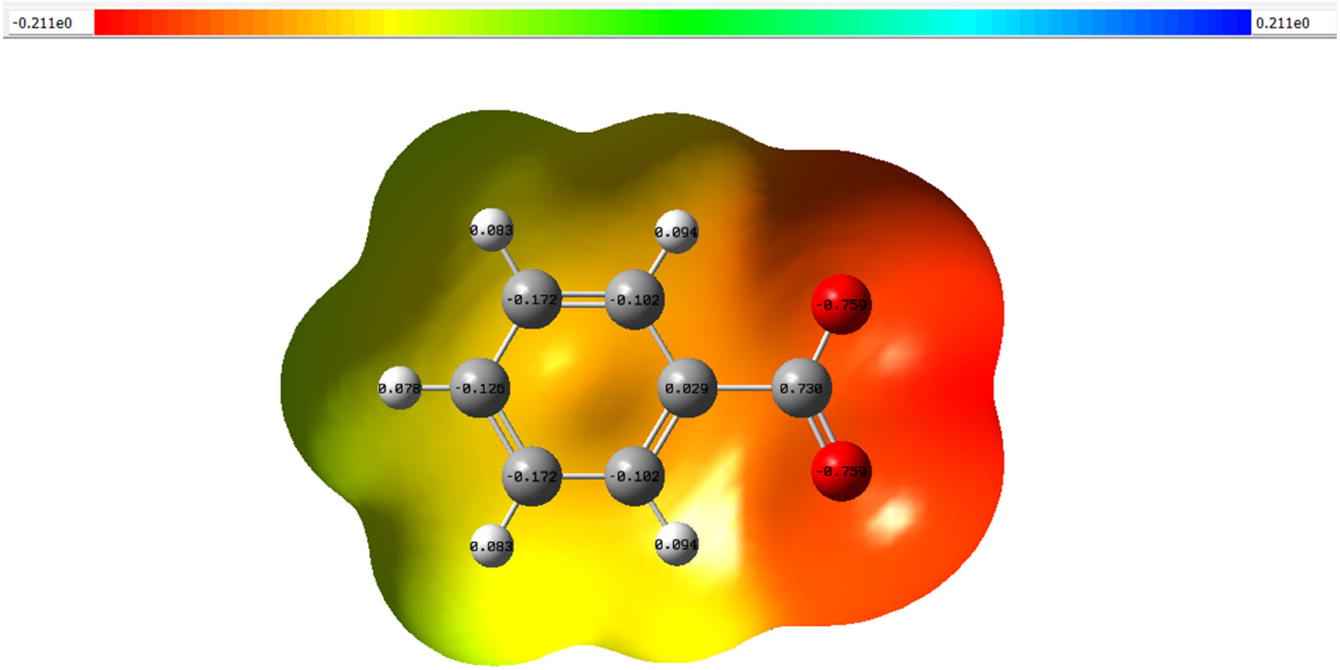
-0.211e0
0.211e0
0.083
0.094
-0.172
-0.102
-0.759
0.078
-0.126
0.029
0.730
-0.172
-0.102
-0.759
0.083
0.094

(c)

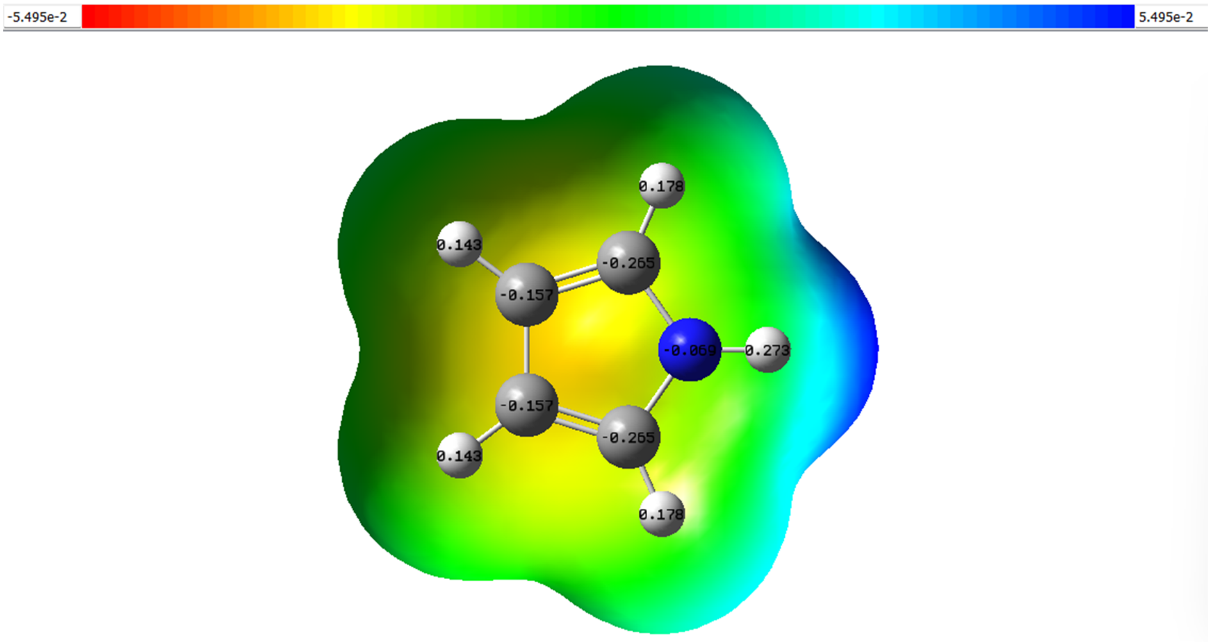

(d)

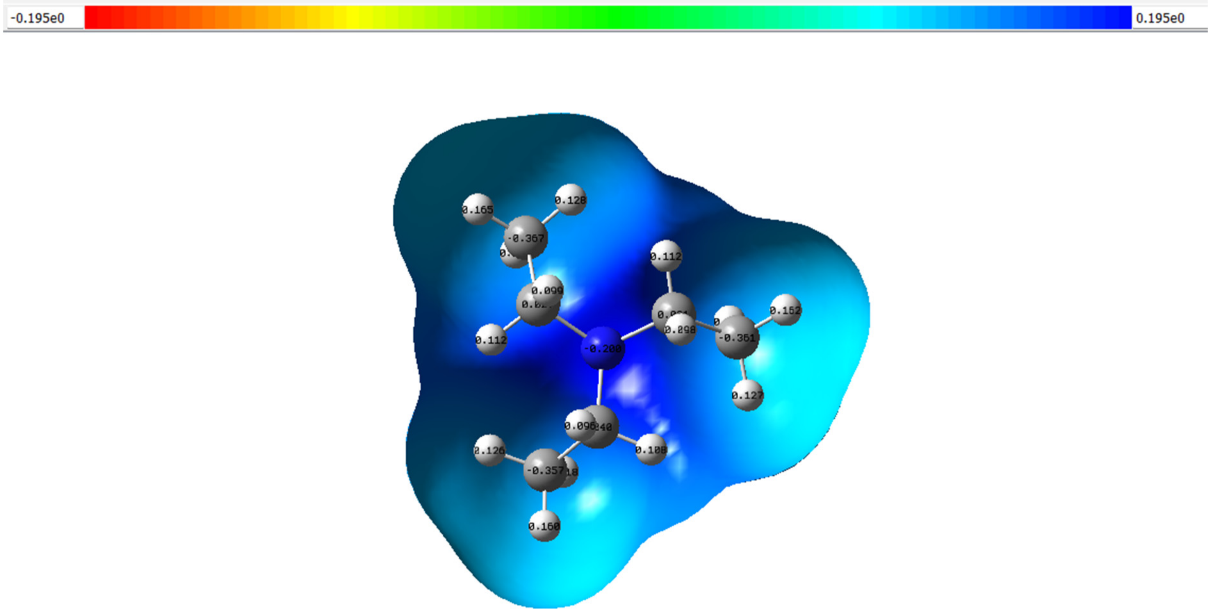

(e)

Figure S2 Electrostatic Potential Surfaces of Anion: (**a**) SA, (**b**) TSA (**c**) BZ, (**d**) pyrrole and (**e**) TEA Optimized at the B3LYP/6-311G + (2d,p) level.
